# Across the Channel and Through Time: Did Lesser Horseshoe Bats Colonise Pantelleria From Europe or North Africa?

**DOI:** 10.1002/ece3.72308

**Published:** 2025-10-10

**Authors:** Luca Cistrone, Emiliano Mori, Mariella Baratti, Mourad Ahmim, Simona Todaro, Andrea Viviano, Danilo Russo

**Affiliations:** ^1^ Laboratory of Animal Ecology and Evolution (AnEcoEvo), Dipartimento di Agraria Università degli Studi di Napoli Federico II Portici (Napoli) Italy; ^2^ Research Institute on Terrestrial Ecosystems (IRET) National Research Council of Italy (CNR) Sesto Fiorentino Italy; ^3^ National Biodiversity Future Center (NBFC) Palermo Italy; ^4^ Ecology and Environment Research Laboratory, Faculty of Natural and Life Sciences University of Bejaia Béjaïa Algeria; ^5^ Department of Earth and Marine Sciences University of Palermo Palermo Italy

**Keywords:** colonisation, island, Maghreb, Pantelleria, Rhinolophid, Sicilian Channel

## Abstract

Islands provide unique opportunities to study historical biogeography, acting as both cradles of endemism and active corridors for species dispersal. The Sicilian Channel, which separates Sicily from the Maghreb, exemplifies this complexity. We investigated the colonisation history of the lesser horseshoe bat ( *Rhinolophus hipposideros* ) on Pantelleria Island (southern Italy) to assess whether its population derives from Europe or North Africa. Because *R. hipposideros* has limited dispersal ability and is largely sedentary, its occurrence on Pantelleria raises questions about past connectivity across the Channel. We analysed mitochondrial markers (COI, cyt‐b, 12S) from Pantelleria, Malta, Algeria, and across the species' range. Phylogenetic and haplotype network analyses place Pantelleria and Malta in a well‐supported clade sister to North African lineages and distinct from European populations. Time‐calibrated analyses based on cyt‐b suggest that the Pantelleria–Malta group diverged from North African conspecifics around 200,000 years ago (MIS 7.2). A palaeogeographical reconstruction for this interval indicates lowered sea level reduced the marine gap between Pantelleria and Tunisia to about 68 km, consistent with over‐sea colonisation from North Africa. The short available sequences for conspecific European bats suggest considering these inferences as provisional. Within these limitations, our results are most consistent with a Maghrebian origin for Pantelleria's *R. hipposideros* , while alternative routes (including European sources) cannot be excluded. Broader genomic sampling, especially from Sicily and Morocco, will be required to resolve colonisation direction and fully establish the population's biogeographic history. More generally, our findings reinforce the view of the Sicilian Channel as an asymmetrically permeable biogeographic corridor that can facilitate faunal exchange across the central Mediterranean.

## Graphical abstract

We investigated the biogeographic origin of the lesser horseshoe bat ( *Rhinolophus hipposideros* ) on Pantelleria Island, a key Mediterranean crossroads. Using mitochondrial DNA, divergence dating and palaeogeographical reconstruction, we found that Pantelleria bats share a recent evolutionary history with Malta and North African lineages, distinct from European populations. These results do not reject a Maghrebian origin via Pleistocene overseas colonisation, highlighting the Sicilian Channel as an asymmetrically permeable biogeographic corridor for this species.

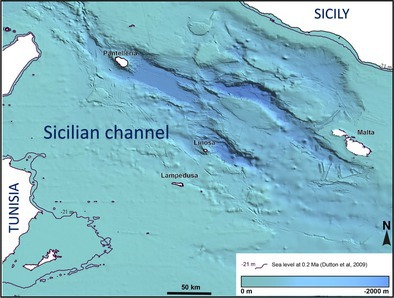

## 1 Introduction

Since Charles Darwin's (1859) revolutionary insights, islands have fascinated biogeographers because of their unique ability to preserve, isolate, and reveal the historical marks of evolutionary divergence (Emerson 2002). Their isolation, limited size, ecological simplicity and well‐defined geological histories make them ideal natural laboratories for studying the origins, dispersal routes and diversification of species (Losos and Ricklefs 2009). Unlike continental environments, islands often show clear patterns of endemism and ecological filtering (Matthews and Triantis 2021; Schrader et al. 2024), providing valuable insights into both ancient vicariant events and more recent colonisation processes.

Although traditionally regarded as isolated systems, islands can also serve as dynamic biogeographic crossroads, connecting different continental faunas and floras over time (Médail 2022). Their strategic location at the junction of tectonic plates, oceanic currents, and migratory routes allows them to function as both stepping‐stones for species dispersal and refuges for relict lineages (Palombo 2018). This is especially evident in regions where major biogeographic realms meet, such as the Mediterranean Basin, where historical and ongoing biotic exchanges have shaped highly diverse and complex communities (Blondel and Aronson 1999). These transitional zones are ideal for studying how geographic proximity, climatic gradients, and geological history influence the composition and evolutionary paths of island biotas (Dapporto et al. 2014). Islands situated within these biogeographic corridors therefore offer valuable insights into the mechanisms behind species colonisation, turnover and persistence across continental boundaries. Furthermore, biogeographic crossroads are areas of significant conservation importance, where intersecting lineages, evolutionary processes and ecological diversity converge, making them especially crucial for protecting both biodiversity and the processes that generate it (Spector 2002).

An excellent example of this intersectional role is the Sicilian Channel, a broad, shallow shelf geologically part of the African Plate, separating Europe from North Africa (Civile et al. 2014). Its islands—Pantelleria, Lampedusa, Linosa, Malta and Gozo—are embedded within the Pelagian Block, a tectonic foreland of the African Plate, yet lie near the European mainland (Jongsma et al. 1985). This unique positioning has made them recipients of colonisation waves from both the African and European mainland, resulting in biotas that reflect a mosaic of Maghrebian, European, and endemic elements. Their complex and restless geological histories, marked by episodic connectivity and isolation due to sea‐level fluctuations, tectonic uplift and subsidence, have mediated colonisation dynamics across time and taxa.

Evidence from various organismal groups confirms the region's role as a dynamic biogeographic corridor. Phylogeographic studies of amphibians and reptiles suggest multiple colonisation events from North Africa to Sicily and its satellite islands during the Pliocene, Pleistocene and Holocene, facilitated by stepping‐stone islands like Pantelleria or temporary land connections (Stöck et al. 2016). The green toad *Bufotes boulengeri* exhibits close mitochondrial and nuclear affinities with its North African relatives, with divergence dating back to between 0.63 and 3.5 million years ago—after the Messinian salinity crisis—indicating that post‐Messinian colonisation likely occurred via terrestrial corridors during sea‐level lowstands (Stöck et al. 2008). Similarly, Sicilian and Tunisian populations of the plant *Ambrosina bassii* exhibit close affinities despite the species' limited dispersal capacity, supporting the hypothesis of a former land bridge between the two areas (Troia et al. 2012).

Island‐specific patterns further underscore the region's complexity. For instance, Pantelleria's butterfly fauna is dominated by North African lineages of *Polyommatus celina*, *Lycaena phlaeas* and *Lasiommata megera*, despite its proximity to Sicily during glacial periods, suggesting asymmetric colonisation routes and the potential for local extinction followed by recolonisation from Tunisia (Vodă et al. 2016). Fossil and faunal evidence from Sicily itself attests to repeated assemblage turnovers and colonisation waves from both Africa and Europe, mediated by land bridges, over‐sea dispersal, or human activity (Palombo 2018). During the Miocene, Sicily belonged to an African paleobioprovince, and later Pleistocene glaciations intermittently exposed land corridors facilitating the arrival of taxa with poor dispersal abilities, such as dwarf elephants (*Palaeoloxodon falconeri*) and small mammals.

Together, these findings portray the Sicilian Channel Islands not as static isolates but as active players in Mediterranean biogeography, where tectonics, sea‐level dynamics, and geographic positioning have influenced a long and intricate history of colonisation, differentiation, and faunal exchange.

While species with clear North African affinities unambiguously indicate south‐to‐north colonisation across the Sicilian Channel, the biogeographic origins of other taxa shared between the Maghreb and Sicily remain unresolved. These cases, where lineages span both sides of the Channel but lack clear phylogeographic directionality, highlight the need for more detailed assessments of colonisation histories. Disentangling the origin and direction of these biotic exchanges is not only vital for reconstructing the region's complex biogeographic history but also essential for understanding how various dispersal pathways, barriers and temporal windows influence island biodiversity. Furthermore, clarifying these patterns has direct implications for conservation: identifying whether insular populations are remnants of African lineages, European expansions, or independent endemics affects how we assess their uniqueness, evolutionary importance and management priorities. In this context, we investigate the biogeographic origin of the lesser horseshoe bat ( *Rhinolophus hipposideros* ) population on Pantelleria Island—a species occurring on both sides of the Sicilian Channel (Schofield et al. 2023). Like other islands in the region (Mifsud and Vella 2019a, 2019b; Gili et al. 2025), Pantelleria hosts bat species of unmistakably Maghrebian origin, alongside others whose colonisation histories remain unresolved and may involve sources from either the African or European mainland (Ancillotto et al. 2020; Fichera et al. 2022; Cistrone et al. 2024, 2025). Bats are generally effective island colonisers due to their ability to fly across open water (Conenna et al. 2017), yet *R. hipposideros* has limited flight capacity and is considered largely sedentary (Schofield et al. 2023). In this study, we apply molecular tools to infer the colonisation route and estimate its timing, combining these results with paleogeographic reconstructions of the Sicilian Channel to understand the mechanisms that enabled this biogeographic event.

Considering the evolutionary history of Pantelleria and the presence of numerous biogeographical and ecological elements of North African origin, we hypothesise the occurrence of a lineage with stronger affinities to North African populations. Specifically, we predict *R. hipposideros* from Pantelleria to be genetically more closely related to conspecifics from North Africa than to those from Europe. This pattern would be consistent with a colonisation scenario from the African continent, supporting the idea that the insular populations originated via dispersal or gene flow across the Sicilian Channel during favourable climatic or geological periods.

## 2 Materials and Methods

### 2.1 Bat Sampling

We captured and sampled *R. hipposideros* individuals on the island of Pantelleria (Italian Ministry of Environment permit number: 0064132.04‐04‐2024) from May to September 2023. We deployed mist nets strategically over water sources, along likely flight paths within wooded areas, and near potential roosting sites. Upon capture, we identified individuals through visual inspection and recorded their sex, age class and reproductive condition. Using a digital calliper and precision scale, we collected morphometric data. We then took a 3‐mm wing membrane biopsy with a sterile punch and immediately preserved each sample in absolute ethanol for later molecular analysis. All bats were released at the capture site within 30 min.

### 2.2 Genetic Analyses

We extracted total genomic DNA from *Rhinolophus hipposideros* individuals sampled in Pantelleria (*n* = 7) and Algeria (*n* = 10) (Table S1) using the Qiagen Blood and Tissue Kit (Qiagen Inc., Tokyo, Japan), following the manufacturer's protocol.

We amplified a 658 bp fragment at the 5′ end of the cytochrome oxidase I (COI) gene—the ‘Folmer region’ – using the universal primers HCO 2198 (5′‐TAAACTTCAGGGTGACCAAAAAATCA‐3′) and LCO 1490 (5′‐GGTCAACAAATCATAAAGATATTGG‐3′) (Folmer et al. 1994). For *R. hipposideros* only, we also amplified a ~300 bp fragment of the cytochrome b (cyt‐*b*) gene using the primers CYTB L14841 (5′‐AAAAAGCTTCCATCCAACATCTCACATGATGAAA‐3′) and CYTB H15149 (5′‐AAACTGCAGCCCCTCAGAATGATATTTGTCCTCA‐3′) (Kocher et al. 1989), as well as a ~390 bp fragment of the 12S gene using primers 12S L1091 (5′‐AAAAGCTTCAAACTGGGATTAGATACCCCACTAT‐3′) and 12S H1478 (5′‐TGACRGCAGAGGGTGACGGGCGGTGTGT‐3′) (Kocher et al. 1989). We selected these three gene regions to compare Pantelleria bats with the widest possible range of conspecifics across the species' distribution (Table S1).

We performed PCR reactions on an Eppendorf MasterCycler X50 thermal cycler in 25 μL volumes containing 100 ng of genomic DNA, 10× buffer, 2 mM MgCl_2_, 200 μM dNTPs, 0.2 μM of each primer, and one unit of Taq polymerase (Life Technologies, Waltham, MA, USA). The thermal cycling protocol included an initial denaturation at 94°C for 5 min, followed by 35 cycles of denaturation at 94°C for 45 s, annealing at 50°C (55°C for 12S) for 30 s, and extension at 72°C for 1 min, with a final extension at 72°C for 10 min (Mori et al. 2024). We visualised PCR products on 1.5% agarose gels stained with 0.5 mg/mL SYBR Safe and purified successful amplifications using the ExoSAP‐IT PCR Clean‐up Kit (Applied Biosystems, Foster City, CA, USA). Sequencing was performed using the Sanger method at BMR Genomics (Padua, Italy; https://www.bmr‐genomics.it). We manually corrected and aligned sequences using MEGA XI (Tamura et al. 2021).

We calculated nucleotide diversity (π), haplotype diversity (h), and the number of polymorphic sites with DNAsp v6.12.03 (Rozas et al. 2017). We aligned our sequences with those previously published in GenBank and BOLD for *R. hipposideros* (accessed April 15, 2025; Table S1 and Figure S1). We inferred phylogenetic relationships using both Bayesian Inference (BI) and Maximum Likelihood (ML). The BI analysis was conducted in MrBayes v3.2.6 (http://mrbayes.sourceforge.net/download.php), running four Markov chains in two independent analyses for 10 million generations, sampling every 1000 generations, and discarding the first 25% as burn‐in. We report posterior probabilities (pp) as node support. ML analyses were carried out in SeaView v5 (Gouy et al. 2021) with 1000 bootstrap replicates.

We selected the most appropriate substitution models and performed tree‐searching operations using Nearest‐Neighbour Interchange (NNI) and Subtree Pruning–Regrafting (SPR) algorithms. Model selection was performed using the Bayesian Information Criterion in MEGA XI, and data were partitioned by gene fragment (for cyt‐b and COI, Tamura‐Nei; for 12S, Kimura‐2‐p). For cyt‐b, we also partitioned by codon position to account for rate heterogeneity across sites. We visualised and edited the resulting phylogenetic trees in FigTree v1.4 (www.tree.bio.ed.ac.uk/software/figtree; University of Edinburgh, UK). To root the trees, we included sequences from *Rhinolophus luctus* (GenBank accession numbers: HM541590, KC747668, OQ511264) as outgroups. All sequences retrieved from GenBank were trimmed to uniform lengths before analysis. The total number of sequences analysed for each gene and each population is reported in Table 1 and in all figure captions for transparency.

To infer genealogical relationships among DNA haplotypes, we employed statistical parsimony analysis following Templeton et al. (1992) and Clement et al. (2002), which estimates the most probable connections between haplotypes based on a 95% parsimony criterion. We generated median joining (MJ) networks for each gene separately using the software ‘Hapsolutely’ (Vences et al. 2021, 2024). We preferred MJ over TCS because MJ is well suited to intraspecific datasets with potential homoplasy/reticulation and is not constrained by a fixed connection limit, providing stable networks for our data (Cassens et al. 2005; Salzburger et al. 2011; Paradis 2018). Separate analyses were conducted for the COI, cyt‐*b* and 12S datasets, as the available sequence data varied across genes in public repositories. MJ networks can handle datasets featuring genetic variation better than other networks (Cassens et al. 2005).

### 2.3 Molecular Clock

We set up the divergence time estimation in BEAUti and ran analyses in BEAST v10.5.0‐beta5 (Suchard et al. 2018). For the cyt‐*b* gene, we followed the analytical framework used by Stoffberg (2007), Liu et al. (2016) and Demos et al. (2019). We applied a strict molecular clock and a Yule speciation process (Yule 1925; Gernhard 2008), using a substitution rate of 3.5% per million years for cyt‐*b*, as previously calibrated for bats on Mediterranean islands (Juste et al. 2004). A strict clock was chosen because all sequences belonged to the same species and exhibited low among‐lineage rate variation, making rate heterogeneity models unnecessary. The substitution model used matched the one identified in the phylogenetic reconstruction step (see above). We ran three independent BEAST analyses, each for 20 million generations, sampling parameters and trees every 1000 generations. We assessed convergence and effective sample sizes (ESS > 200) using Tracer v1.7.2. We used LogCombiner to discard the first 25% of samples as burn‐in and merge the output files. Finally, we used TreeAnnotator to generate the maximum clade credibility tree with 95% highest probability densities.

### 2.4 Palaeogeographical Reconstruction

To assess potential colonisation pathways to Pantelleria during the Middle Pleistocene, we reconstructed the palaeogeographical configuration of the Sicilian Channel at approximately 200,000 years ago, corresponding to Marine Isotope Stage 7.2. A high‐resolution digital elevation model was generated using bathymetric data from the European Marine Observation and Data Network (EMODnet: http://www.emodnet‐bathymetry.eu). A palaeo‐sea‐level estimate of −21 m was applied, based on U–Th dated records from submerged speleothems. Areas of the seafloor with present‐day depth shallower than −21 m were considered as potentially emergent during MIS 7.2. Pleistocene sea level fluctuated, but our reconstruction pertains to MIS 7.2 (−21 m) only and should not be extrapolated to other stages. To ensure the reliability of the reconstruction, we considered only regions of the Sicilian Channel known to be tectonically stable since the Middle Pleistocene. Seafloor morphological features, including islands, seamounts, ridges and banks, were visually assessed, and distances between Pantelleria and potential source areas (Tunisia, Linosa, Lampedusa, Malta) were recalculated based on the adjusted sea‐level scenario.

## 3 Results

### 3.1 Genetic Diversity and Phylogenetic Relationships

We successfully amplified all gene fragments. Nucleotide diversity (π), haplotype diversity (h), and number of polymorphic sites are shown in Table 1.

**TABLE 1:** Nucleotide diversity (π), haplotype diversity (h) and number of polymorphic sites of all analysed genes.

Species	gene fragment	Haplotypes in our sample	Haplotypes from GenBank	Π	h	*N* polymorphic sites	*N* sequences
*R. hipposideros*	COI	8	6	0.01	0.83	45	30
*R. hipposideros*	cyt‐*b*	3	11	0.01	0.71	14	112
*R. hipposideros*	12S	3	1	0.01	0.71	9	25

**FIGURE 1:**
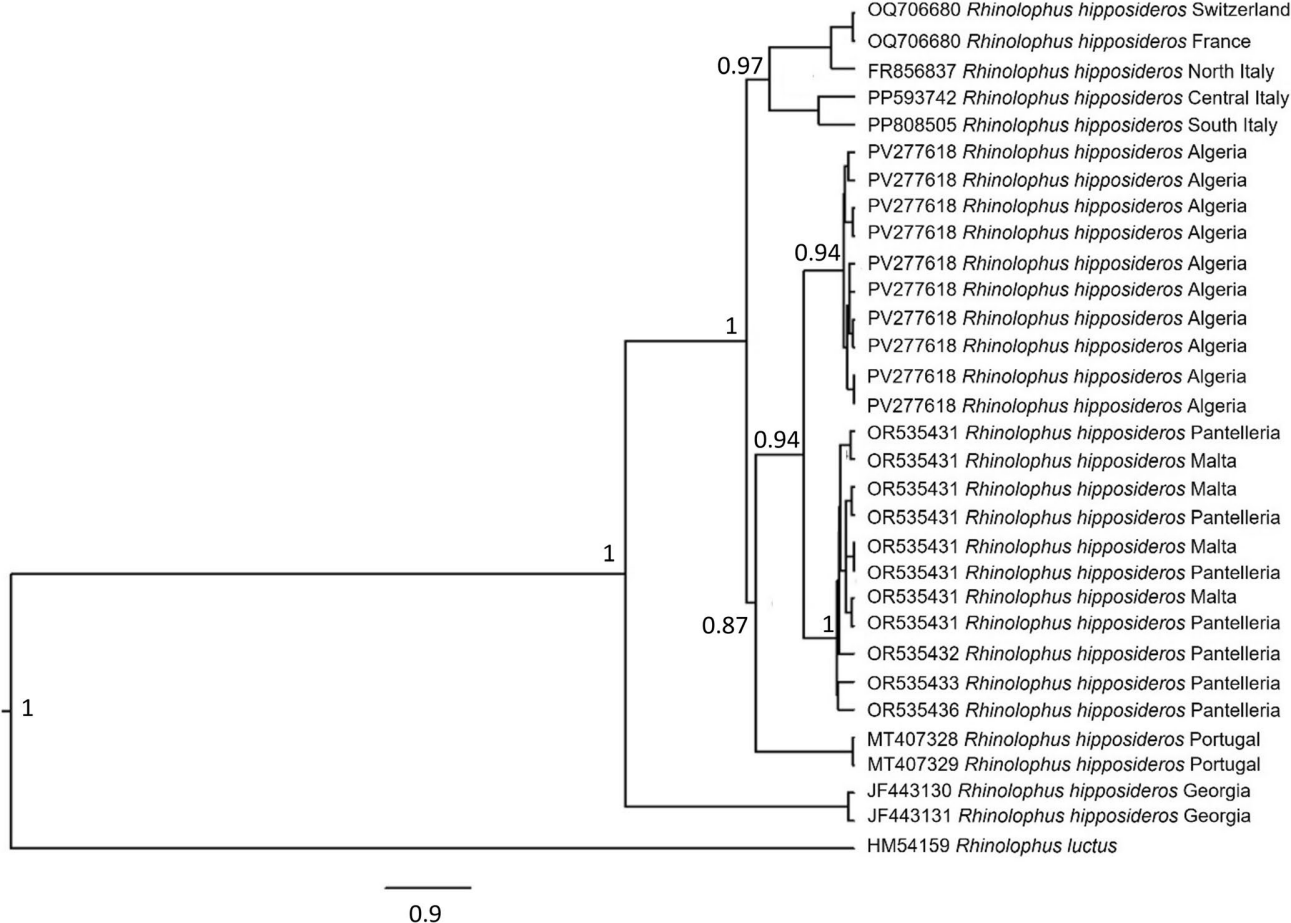
Bayesian phylogenetic relationships of *Rhinolophus hipposideros* using COI sequences (*N* = 30 sequences). Bootstrap values for Bayesian reconstructions are indicated at tree nodes. Values < 50% are not shown. The same accession numbers refer to different individuals who share the same haplotype for this gene. Samples from Northern, Central and Southern Italy, respectively, originated from Lombardy, Tuscany and Apulia (Figure S2).

**FIGURE 2:**
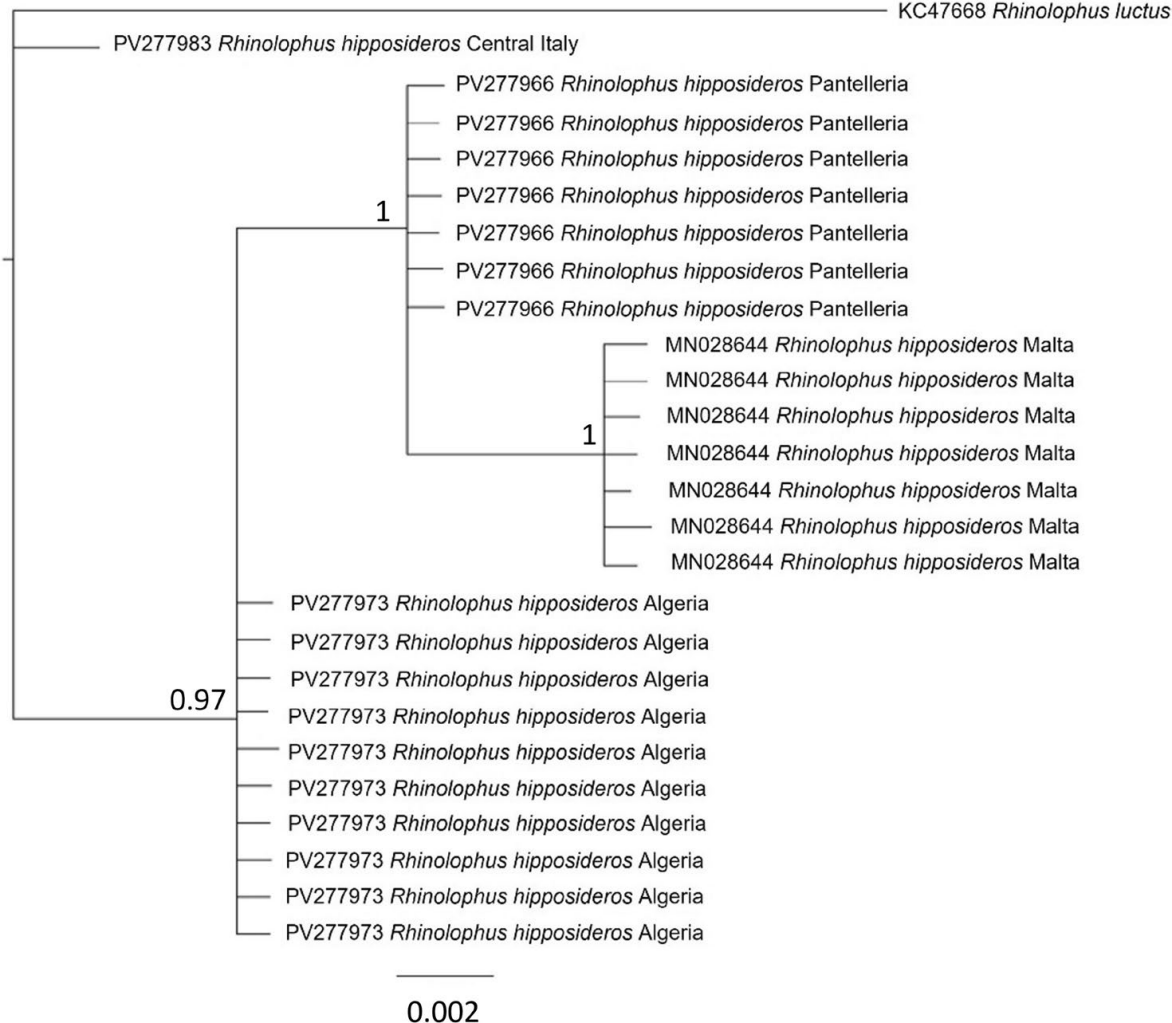
Bayesian phylogenetic relationships of *Rhinolophus hipposideros* using 12S sequences (*N* = 25 sequences). Bootstrap values for Bayesian reconstructions are indicated at tree nodes. Values < 50% are not shown. The same accession numbers refer to different individuals sharing the same haplotype for this gene. The sample from Central Italy was collected in Tuscany (Figure S2).

**FIGURE 3:**
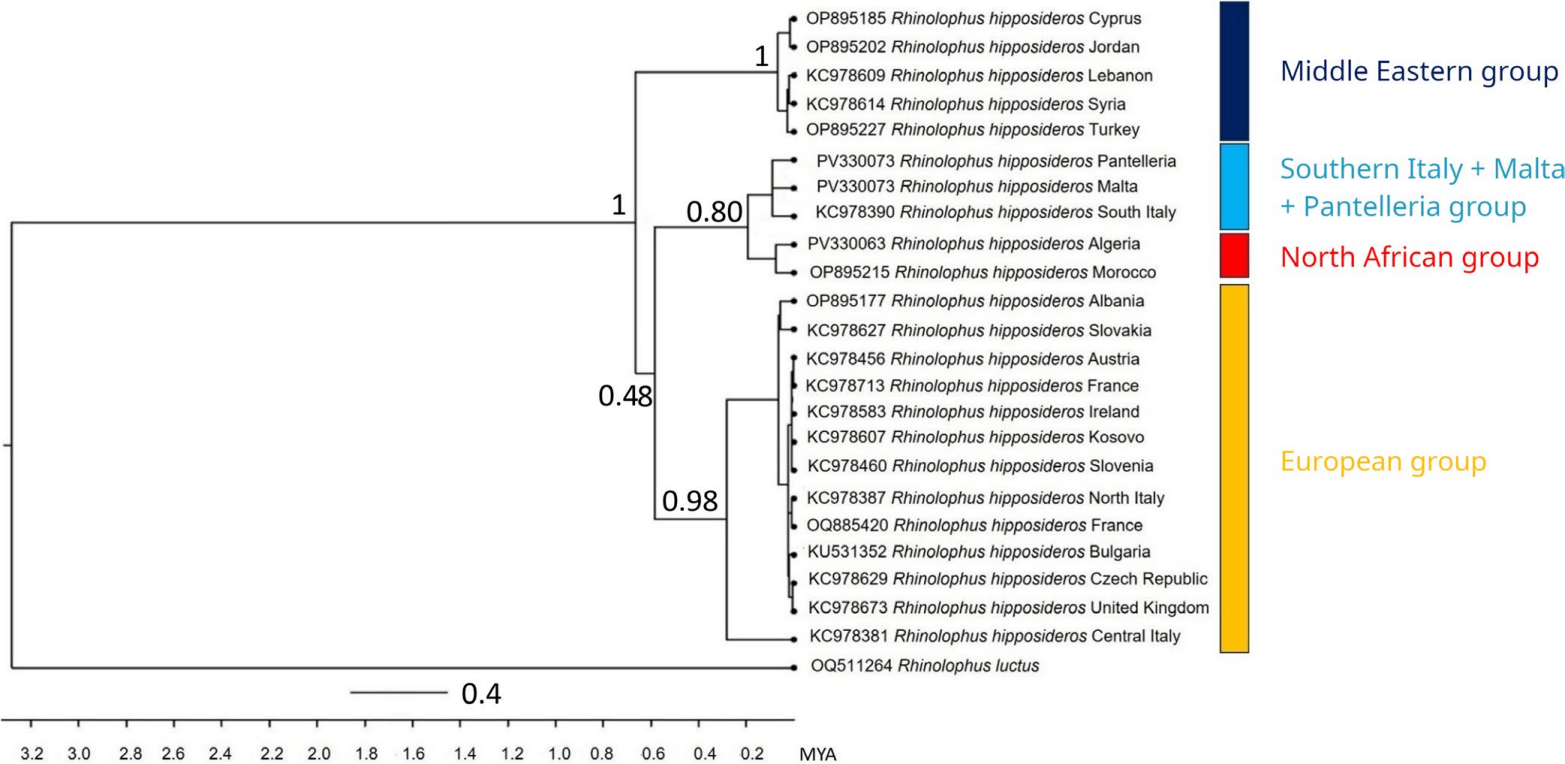
Time‐scaled tree inferred by BEAST of *Rhinolophus hipposideros* using cyt‐*b* sequences (N_tot_ = 117 sequences, but only 23 are shown in the tree); 95% HPD credibility intervals for each well‐supported recovered node are represented by blue horizontal lines. Posterior probabilities (PP) are reported for each node (values < 0.8 are not shown). Samples from Northern, Central and Southern Italy were collected respectively in Lombardy, Tuscany and Campania (Figure S2). MYA = million years ago.

For all genes, samples of *R. hipposideros* from Pantelleria and Malta clustered together and were the sister group of North African conspecifics (Figures 1, 2, 3).

**FIGURE 4:**
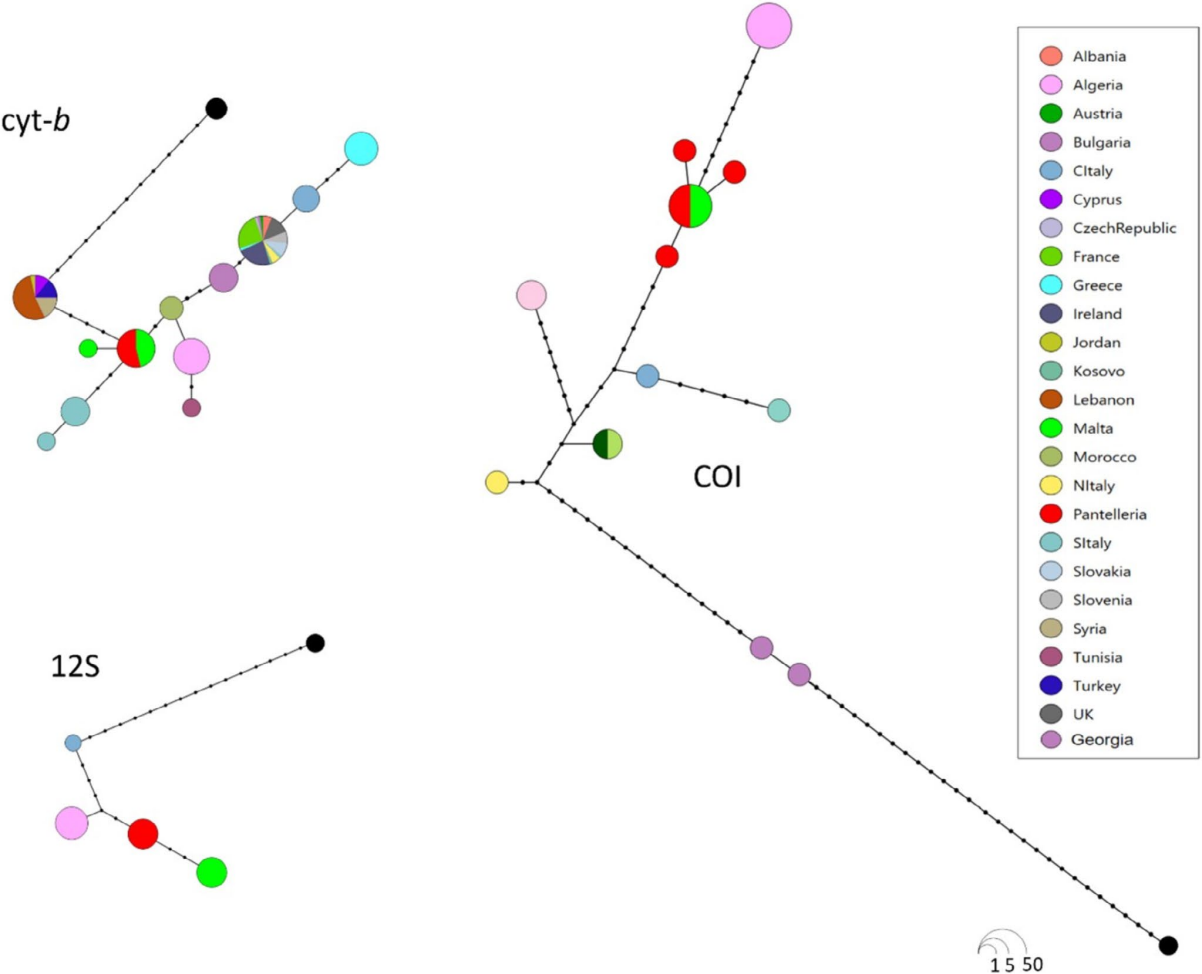
MJ network showing the relationship among COI (*N* = 30 sequences), 12S (*N* = 25 sequences) and cyt‐*b* (*N* = 117 sequences) haplotypes of *R. hipposideros* . Circles represent different haplotypes. Circle sizes are proportional to the number of individuals examined for each haplotype. Dashes indicate more than one mutational step between linked haplotypes. The black filled circle corresponds to the outgroup *Rhinolophus luctus* .

The same patterns were shown by the MJ haplotype networks (Figure 4).

The phylogenetic tree based on the cyt b gene shows a clear division between the populations of *R. hipposideros* of the Middle Eastern Mediterranean and North Africa and those of central and western Europe, with island populations (e.g., Malta, Pantelleria) genetically close to the North African ones. However, the low support at the main node (0.48) dictates caution in interpreting this bifurcation. Divergence‐time analyses based on cyt‐b indicate that the Middle Eastern *R. hipposideros* lineage diverged from all others ca. 700,000 years ago. The North African lineage and the Pantelleria–Malta clade split approximately 200,000 years ago, whereas the combined North African–Pantelleria–Malta group diverged from the European lineage around 550,000 years ago (Figure 3). The consistency between high Bayesian support values and narrow HPD ranges at the end nodes (Figure 3) suggests good topology reliability at the intra‐clade level, while nodes with low support show a certain degree of uncertainty about both the topology and the history of divergence events.

### 3.2 Palaeogeographical Reconstruction

**FIGURE 5:**
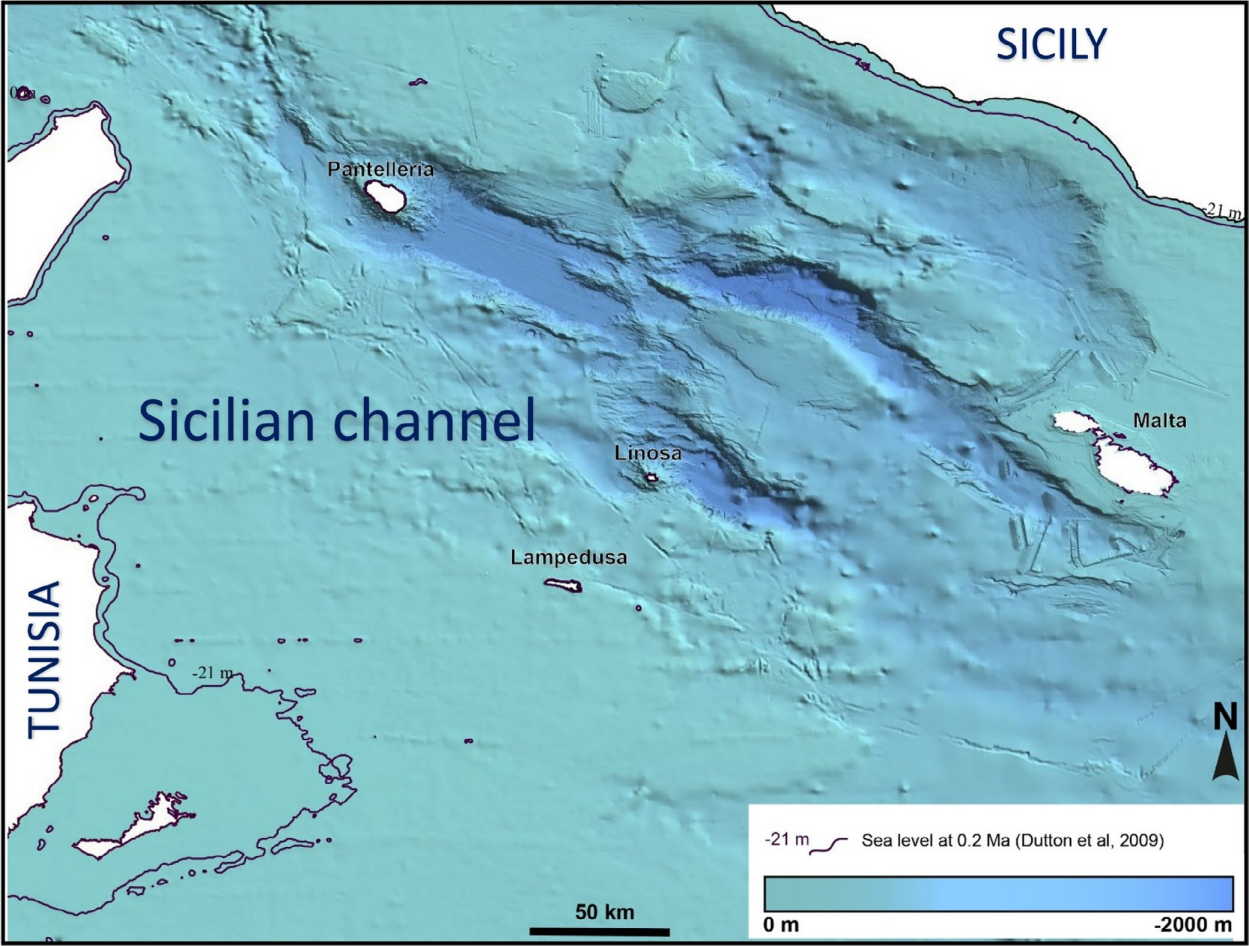
Palaeogeographical reconstruction of the Sicilian Channel at approximately 200,000 years ago (Marine Isotope Stage 7.2), based on a sea‐level drop of −21 m (purple contour; Dutton et al. 2009). The contour delineates areas that would have emerged above sea level, substantially narrowing marine gaps between Tunisia, Pantelleria, Linosa, Lampedusa and Malta. Bathymetry is shown in shades of blue (0 to −2000 m).

The application of a −21 m sea‐level offset resulted in a significant increase in emerged land across the Sicilian Channel. Under these conditions, the shortest marine gap between Pantelleria and the North African coast was reduced to approximately 68 km (current distance = 85 km), versus 100 km separating Pantelleria from Sicily (currently, 105 km) (Figure 5). The distance between Lampedusa and Linosa was (and currently is) 43 km, while Linosa lay 121 km from Pantelleria and 132 km from Malta. No additional emergent landforms or seamounts were identified within the channel under the MIS 7.2 sea‐level scenario. The seafloor morphology surrounding Pantelleria remained steep and isolated, and the 21 m sea‐level drop did not substantially alter distances to nearby islands.

## 4 Discussion

We provide new insights into the biogeographic history of *R. hipposideros* on Pantelleria, which, although interesting, must be interpreted cautiously. Several factors limit the strength of our inferences, including the use of relatively short mitochondrial fragments, the absence of nuclear data, the reliance on a substitution rate estimated for another bat species, incomplete geographic sampling (especially from Sicily and Morocco), and limited resolution for some gene regions such as 12S. Moreover, mitochondrial phylogenies alone cannot establish colonisation direction with certainty, and the near‐simultaneous divergence of multiple lineages leaves open the possibility of alternative scenarios. This caution accords with the general point that sister relationships and apparent ‘basal’ positions do not, by themselves, establish ancestor–descendant relationships (Crisp and Cook 2005). These limitations should be considered when interpreting the results presented below. At the same time, a strength of our approach is the integration of three mitochondrial markers with a time‐calibrated phylogenetic framework and an independent MIS 7.2 palaeogeographical reconstruction, which together provide a coherent basis for evaluating alternative colonisation scenarios.

By combining mitochondrial sequence data from three gene fragments with time‐calibrated phylogenetic reconstructions, we found that this insular population shares a recent evolutionary history with conspecifics from Malta and is phylogenetically distinct from European populations. Notably, the COI and Cytb phylogenies showed higher resolution than 12S, probably due to the inclusion of a broader geographical sampling. In contrast, the limited representation of sequences from Europe and Northern Africa in the 12S dataset restricted its ability to clarify the origin of populations in the Sicilian Channel. Importantly, molecular clock analyses indicate that the Pantelleria‐Malta clade diverged from the North African lineage around 200,000 years ago, a pattern most consistent with, but not conclusive for, a Maghrebian rather than European origin.

We analysed a short but highly variable fragment of the Cyt‐b gene, as this region was the most widely available in GenBank and allowed us to maximise both sample size and geographic coverage. We acknowledge that longer sequences would provide higher resolution and that the short length of Cyt‐b might limit diversity estimates and slightly affect divergence‐time precision. We also recognise that applying a substitution rate from a different bat species may introduce some bias into absolute divergence‐time estimates. Nevertheless, because we targeted the most variable region of the gene and included the maximum number of sequences available, we consider the overall phylogenetic and temporal patterns robust enough to provide meaningful insights into the species' evolutionary history, while noting that future work with full‐length sequences and genomic data would help refine these results. Finally, the absence of nuclear data may have provided only a partial perspective, which future analyses should aim to refine. Previous phylogeographic research on *R. hipposideros* has revealed a complex history driven by survival in multiple glacial refugia across the Mediterranean region (Dool et al. 2013). Using mitochondrial and nuclear markers, Dool et al. (2013) identified distinct lineages in Morocco, Tunisia, Southern Italy, Malta and the Balkans, indicating long‐term persistence rather than recent colonisation. Notably, Malta and Southern Italy were identified as independent glacial refugia, with no evidence that they were recolonised from northern or central Europe after the Last Glacial Maximum (LGM). Moreover, *R. hipposideros* shows reduced dispersal capacity and strong philopatry (Dool et al. 2013), consistent with the limited role of long‐distance postglacial expansions in shaping current diversity patterns. In this context, the estimated divergence time of around 200,000 years between the Pantelleria‐Malta and Maghrebian lineages is particularly significant. This period predates the LGM (~20,000 years ago) and makes a colonisation from Europe via Sicily during postglacial times less likely, but it cannot be entirely excluded. Data from Sicily are currently lacking, but existing evidence suggests that Pantelleria bats are unlikely to have originated from Italian or broader European lineages. Although alternative routes cannot be ruled out, a Maghrebian origin, potentially through direct colonisation from Tunisia across the Sicilian Channel during a Pleistocene interglacial period when sea levels were lower, appears more plausible.

The biogeographic pattern observed in *R. hipposideros* on Pantelleria is consistent with the Sicilian Channel acting as a dynamic faunal corridor, where dispersal and isolation have likely interacted to shape insular diversity. This case adds to growing evidence suggesting that the Channel's permeability has been asymmetric and strongly context‐dependent over time (e.g., Stöck et al. 2008, 2016; Corso et al. 2012; Troia et al. 2012; Vodă et al. 2016; Muscarella and Baragona 2017; Palombo 2018). Our palaeogeographical reconstruction of the Sicilian Channel at MIS 7.2 is consistent with the feasibility of such a scenario. During this period, a relative sea‐level lowstand of approximately 21 m below present (Dutton et al. 2009) expanded emergent land and narrowed marine gaps between landmasses. Under these conditions, the shortest marine crossing between Pantelleria and Tunisia would have been reduced to approximately 68 km (from the current 85 km), and the distance to Sicily would have remained over 100 km. While still substantial, this distance falls within the dispersal capabilities of bats and appears more plausible than alternative routes involving stepping‐stone islands. The reconstructed distances from Pantelleria to Linosa (121 km, similar to the present) and Malta (via Linosa, over 250 km total, with the current distance lower than 210 km) were considerably longer, and no intermediate seamounts were exposed at that time (Civile et al. 2014; Spatola et al. 2018), making the direct Tunisia–Pantelleria route a parsimonious explanation, yet other routes cannot be excluded. The lack of unique haplotypes in Pantelleria suggests limited divergence following colonisation. Moreover, the colonisation timing we estimated indicates that *R. hipposideros* persisted on Pantelleria through major Late Pleistocene volcanic crises, underscoring resilience to extreme environmental disturbance (Rotolo et al. 2013).

Pantelleria's ability to sustain a resident population of *R. hipposideros* likely reflects the island's availability of both historical and present‐day roosting and foraging resources. Today, the species primarily roosts in traditional stone buildings known as ‘dammusi’, which provide stable microclimates suitable for small nursery colonies during the reproductive season (Cistrone et al. 2024). At the time of colonisation, however, the island's abundance of caves and volcanic rock cavities would also have offered ample natural roosting sites. Likewise, while *R. hipposideros* is typically associated with forested habitats for foraging, it exhibits a degree of ecological flexibility, also using scrubland and more open habitats (Schofield et al. 2023) and even deserts (D. Russo, pers. obs.). Although torpor use is influenced by multiple factors, small‐bodied bats inhabiting warm climates tend to enter torpor at higher ambient temperatures and achieve greater metabolic suppression than larger species (Ayala‐Berdon and Medina‐Bello 2024). This interaction between body size and climate may have made torpor an especially effective strategy for *R. hipposideros* on Pantelleria, where seasonal resource fluctuations and mild temperatures favoured its use as an energy‐saving mechanism, potentially critical to post‐colonisation survival.

From a methodological standpoint, our results also underscore the importance of calibrating phylogenetic trees when reconstructing biogeographic scenarios. While uncalibrated phylogenies indicated a superficial clustering of Pantelleria with European haplotypes based on sequence similarity, time‐calibrated analyses offered a markedly different interpretation of the evolutionary relationships and highlighted the true temporal depth of divergence between lineages. This highlights the limitations of relying solely on topological proximity in phylogenies to infer dispersal history and reinforces the need to combine genetic data with divergence dating and palaeogeographical context (Rutschmann 2006; Ho and Phillips 2009; Smedmark et al. 2010; Landis 2017).

Our findings suggest a Maghrebian origin of the *R. hipposideros* population on Pantelleria during a warm Pleistocene phase, with subsequent isolation and potential genetic exchange with Malta (possibly belonging to the *R. h. escalerae* clade: Lanza 2012). This is confirmed by the occurrences of the latest fossil records of *R. hipposideros* from Malta dating back to the lower Pleistocene and Holocene (Storch 1974). The only other species for which genetic data are available from Pantelleria is the Maghrebian bat *Plecotus gaisleri*. This shows that Malta and Pantelleria are more closely related to North Africa and Lampedusa; on the mainland, the species is ecologically replaced by other *Plecotus* species (Gili et al. 2025). In the case of Malta alone, *Pipistrellus kuhlii, Pipistrellus pipistrellus* and *Hypsugo savii* also show stronger affinities with North Africa than with Central Europe (Batsleer et al. 2019).

While a West Asian origin of *R. hipposideros* is well supported (Benda et al. 2022), our results suggest that North African populations, at least in Tunisia and Algeria, were likely colonised from Italy rather than Iberia, and the Moroccan population may have originated from southern Spain. This interpretation is consistent with the genetic affinities between North African and Southern Mediterranean island populations, pointing to a possible colonisation route across the Sicilian Channel, with Europe (the clade showing the earliest divergence) as the source and Pantelleria, Malta, and Lampedusa serving as stepping stones. Pantelleria and Malta, in particular, form sister clades that diverged at the same time. Nevertheless, the Iberian connection proposed by Dool et al. (2013) remains a plausible alternative that requires further sampling, especially in Morocco.

Overall, our findings are most consistent with a Maghrebian origin of the Pantelleria population of *R. hipposideros* , but the evidence remains incomplete. Alternative colonisation routes remain possible given current data limitations. Broader nuclear genomic studies combined with coalescent‐based methods and denser geographic sampling will be essential to resolve colonisation direction and timing more robustly. Such data will also clarify how historical sea‐level fluctuations and habitat availability shaped the dynamics of faunal exchange across the Sicilian Channel. Our results contribute to a growing recognition of the biogeographic importance of the Maghreb and its influence in shaping patterns of genetic diversity across Mediterranean islands (Ancillotto et al. 2020; Fichera et al. 2022; Gili et al. 2025). More broadly, our case reinforces the value of insular systems, particularly those situated at the confluence of major biogeographic realms, as ‘natural experiments’ for understanding historical colonisation and persistence. The Sicilian Channel exemplifies how tectonic, climatic, and ecological variables interact to shape biodiversity patterns across time and space. As biodiversity hotspots and crossroads, such islands are not only key to retracing evolutionary histories but also offer strategic opportunities for conserving lineages that embody distinct biogeographic trajectories.

## Author Contributions

**Luca Cistrone:** conceptualization (equal), formal analysis (equal), investigation (lead), methodology (equal), writing – original draft (equal), writing – review and editing (equal). **Emiliano Mori:** formal analysis (lead), methodology (equal), visualization (equal), writing – review and editing (equal). **Mariella Baratti:** formal analysis (lead), funding acquisition (equal), methodology (lead), visualization (equal), writing – review and editing (equal). **Mourad Ahmim:** investigation (equal), writing – review and editing (equal). **Simona Todaro:** investigation (equal), writing – review and editing (equal). **Andrea Viviano:** formal analysis (equal), writing – review and editing (equal). **Danilo Russo:** conceptualization (lead), formal analysis (equal), funding acquisition (lead), investigation (lead), methodology (equal), supervision (lead), writing – original draft (lead), writing – review and editing (lead).

## Conflicts of Interest

The authors declare no conflicts of interest.

## Supporting information


**Appendix S1:** ece372308‐sup‐0001‐AppendixS1.docx.

## Data Availability

All mitochondrial sequences generated in this study have been deposited in GenBank under the accession numbers listed in Table S1.

## References

[ece372308-bib-0001] Ancillotto, L. , L. Bosso , S. Smeraldo , et al. 2020. “An African Bat in Europe, *Plecotus Gaisleri*: Biogeographic and Ecological Insights From Molecular Taxonomy and Species Distribution Models.” Ecology and Evolution 10: 5785–5800. 10.1002/ece3.6317. 32607190 PMC7319239

[ece372308-bib-0002] Ayala‐Berdon, J. , and K. I. Medina‐Bello . 2024. “Torpor Energetics Are Related to the Interaction Between Body Mass and Climate in Bats of the Family Vespertilionidae.” Journal of Experimental Biology 227, no. 18: jeb246824. 39206564 10.1242/jeb.246824

[ece372308-bib-0003] Batsleer, F. , E. Portelli , J. J. Borg , A. Kiefer , M. Veith , and D. Dekeukeleire . 2019. “Maltese Bats Show Phylogeographic Affiliation With North‐Africa: Implications for Conservation.” Hystrix the Italian Journal of Mammalogy 30, no. 2: 172–177.

[ece372308-bib-0004] Benda, P. , M. Uvizl , P. Vallo , A. Reiter , and M. Uhrin . 2022. “A Revision of the *Rhinolophus hipposideros* Group (Chiroptera: Rhinolophidae) With Definition of an Additional Species From the Middle East.” Acta Chiropterologica 24, no. 2: 269–298. 10.3161/15081109ACC2022.24.2.001.

[ece372308-bib-0005] Blondel, J. , and J. Aronson . 1999. Biology and Wildlife of the Mediterranean Region. Oxford University Press.

[ece372308-bib-0006] Cassens, I. , P. Mardulyn , and M. C. Milinkovitch . 2005. “Evaluating Intraspecific ‘Network’ Construction Methods Using Simulated Sequence Data: Do Existing Algorithms Outperform the Global Maximum Parsimony Approach?” Systematic Biology 54, no. 3: 363–372. 10.1080/10635150590945377. 16012104

[ece372308-bib-0007] Cistrone, L. , A. M. Augusto , G. Fichera , H. Rebelo , and D. Russo . 2024. “Agriculture and Water Availability Show Contrasting Effects on Bats in a Mediterranean Island of Outstanding Chiropteran Biogeographical Value.” Ecology and Evolution 14, no. 12: e70717. 39717639 10.1002/ece3.70717 PMC11664209

[ece372308-bib-0008] Cistrone, L. , H. Schofield , and D. Russo . 2025. “Ground Cover Promotes Enhanced Bat Activity in High‐Value Insular Vineyards.” Agriculture, Ecosystems & Environment 389: 109698.

[ece372308-bib-0009] Civile, D. , E. Lodolo , M. Zecchin , et al. 2014. “The Lost Adventure Archipelago (Sicilian Channel, Mediterranean Sea): Morpho‐Bathymetry and Late Quaternary Palaeogeographic Evolution.” Global and Planetary Change 125: 36–47. 10.1016/j.gloplacha.2014.12.003.

[ece372308-bib-0010] Clement, M. , D. Posada , and K. A. Crandall . 2002. “TCS: A Computer Program to Estimate Gene Genealogies.” Molecular Ecology 9: 1657–1660. 10.1046/j.1365-294x.2000.01020.x 11050560

[ece372308-bib-0011] Conenna, I. , R. Rocha , D. Russo , and M. Cabeza . 2017. “Insular Bats and Research Effort: A Review of Global Patterns and Priorities.” Mammal Review 47, no. 3: 169–182.

[ece372308-bib-0012] Corso, A. , V. Penna , M. Gustin , I. Maiorano , and P. Ferrandes . 2012. “Annotated Checklist of the Birds From Pantelleria Island (Sicilian Channel, Italy): A Summary of the Most Relevant Data, With New Species for the Site and for Italy.” Biodiversity Journal 3, no. 4: 407–428.

[ece372308-bib-0013] Crisp, M. D. , and L. G. Cook . 2005. “Do Early Branching Lineages Signify Ancestral Traits?” Trends in Ecology & Evolution 20, no. 3: 122–128. 16701355 10.1016/j.tree.2004.11.010

[ece372308-bib-0014] Dapporto, L. , S. Fattorini , R. Vodă , V. Dincă , and R. Vila . 2014. “Biogeography of Western Mediterranean Butterflies: Combining Turnover and Nestedness Components of Faunal Dissimilarity.” Journal of Biogeography 41, no. 9: 1639–1650.

[ece372308-bib-0015] Darwin, C. 1859. On the Origins of Species by Means of Natural Selection. Murray Editions.

[ece372308-bib-0016] Demos, T. C. , P. W. Webala , S. M. Goodman , J. C. Kerbis Peterhans , M. Bartonjo , and B. D. Patterson . 2019. “Molecular Phylogenetics of the African Horseshoe Bats (Chiroptera: Rhinolophidae): Expanded Geographic and Taxonomic Sampling of the Afrotropics.” BMC Evolutionary Biology 19: 1–14. 10.1186/s12862-019-1485-1. 31434566 PMC6704657

[ece372308-bib-0017] Dool, S. E. , S. J. Puechmaille , C. Dietz , et al. 2013. “Phylogeography and Postglacial Recolonization of Europe by *Rhinolophus Hipposideros*: Evidence From Multiple Genetic Markers.” Molecular Ecology 22: 4055–4070. 10.1111/mec.12373. 23889545

[ece372308-bib-0018] Dutton, A. , F. Antonioli , and E. Bard . 2009. “A New Chronology of Sea Level Highstands for the Penultimate Interglacial.” Nature Geoscience 17, no. 2: 66–68.

[ece372308-bib-0019] Emerson, B. C. 2002. “Evolution on Oceanic Islands: Molecular Phylogenetic Approaches to Understanding Pattern and Process.” Molecular Ecology 11, no. 6: 951–966. 12030975 10.1046/j.1365-294x.2002.01507.x

[ece372308-bib-0020] Fichera, G. , M. Mucedda , D. Russo , et al. 2022. “Pantelleria Island (Sicily, Italy): A Biogeographic Crossroad for Bats Between Africa and Europe.” Hystrix, the Italian Journal of Mammalogy 33, no. 2: 134–137.

[ece372308-bib-0021] Folmer, O. , M. Black , W. Hoeh , R. Lutz , and R. Vrijenhoek . 1994. “DNA Primers for Amplification of Mitochondrial Cytochrome c Oxidase Subunit I From Diverse Metazoan Invertebrates.” Molecular Marine Biology and Biotechnology 3: 294. 7881515

[ece372308-bib-0022] Gernhard, T. 2008. “The Conditioned Reconstructed Process.” Journal of Theoretical Biology 253: 769–778. 10.1016/j.jtbi.2008.04.005. 18538793

[ece372308-bib-0023] Gili, F. , P. di Bari , M. Massaad , et al. 2025. “Non‐Invasive Survey Techniques Uncover the Coexistence of African and European Bats on the Island of Lampedusa.” Mammalian Biology 105: 533–540.

[ece372308-bib-0024] Gouy, M. , E. Tannier , N. Comte , and D. P. Parsons . 2021. “Seaview Version 5: A Multiplatform Software for Multiple Sequence Alignment, Molecular Phylogenetic Analyses, and Tree Reconciliation.” In Multiple Sequence Alignment: Methods and Protocols, edited by K. Katoh , 241–260. Springer Editions. 10.1007/978-1-0716-1036-7_15 33289897

[ece372308-bib-0025] Ho, S. Y. , and M. J. Phillips . 2009. “Accounting for Calibration Uncertainty in Phylogenetic Estimation of Evolutionary Divergence Times.” Systematic Biology 58, no. 3: 367–380. 10.1093/sysbio/syp035. 20525591

[ece372308-bib-0026] Jongsma, D. , J. E. van Hinte , and J. M. Woodside . 1985. “Geologic Structure and Neotectonics of the North African Continental Margin South of Sicily.” Marine and Petroleum Geology 2, no. 2: 156–179.

[ece372308-bib-0027] Juste, J. , C. Ibáñez , J. Muñoz , et al. 2004. “Mitochondrial Phylogeography of the Long‐Eared Bats (*Plecotus*) in the Mediterranean Palaearctic and Atlantic Islands.” Molecular Phylogenetics and Evolution 31, no. 3: 1114–1126. 10.1016/j.ympev.2003.10.005. 15120404

[ece372308-bib-0028] Kocher, T. D. , W. K. Thomas , A. Meyer , et al. 1989. “Dynamics of Mitochondrial DNA Evolution in Animals: Amplification and Sequencing With Conserved Primers.” Proceedings of the National Academy of Sciences of the United States of America 86: 6196–6200. 10.1073/pnas.86.16.6196. 2762322 PMC297804

[ece372308-bib-0029] Landis, D. A. 2017. “Designing Agricultural Landscapes for Biodiversity‐Based Ecosystem Services.” Basic and Applied Ecology 18: 1–12. 10.1016/j.baae.2016.07.005.

[ece372308-bib-0030] Lanza, B. 2012. Fauna d'Italia–Vol XLVII–Mammalia v–Chiroptera. Edagricole.

[ece372308-bib-0031] Liu, T. , K. Sun , Y. C. Park , and J. Feng . 2016. “Phylogenetic Relationships and Evolutionary History of the Greater Horseshoe Bat, *Rhinolophus ferrumequinum* , in Northeast Asia.” PeerJ 4: e2472. 10.7717/peerj.2472. 27761309 PMC5068396

[ece372308-bib-0032] Losos, J. B. , and R. E. Ricklefs . 2009. “Adaptation and Diversification on Islands.” Nature 457, no. 7231: 830–836. 19212401 10.1038/nature07893

[ece372308-bib-0033] Matthews, T. J. , and K. Triantis . 2021. “Island Biogeography.” Current Biology 31, no. 19: R1201–R1207. 34637732 10.1016/j.cub.2021.07.033

[ece372308-bib-0034] Médail, F. 2022. “Plant Biogeography and Vegetation Patterns of the Mediterranean Islands.” Botanical Review 88, no. 1: 63–129.

[ece372308-bib-0035] Mifsud, C. M. , and A. Vella . 2019a. “Mitochondrial Genetic Diversity of Bat Species From the Maltese Islands and Applications for Their Conservation.” Natural and Engineering Sciences 4, no. 3: 276–292. 10.28978/nesciences.646348.

[ece372308-bib-0036] Mifsud, C. M. , and A. Vella . 2019b. “Acoustic Characterization of Bats From Malta: Setting a Baseline for Monitoring and Conservation of Bat Populations.” Bioacoustics 28, no. 5: 427–442.

[ece372308-bib-0037] Mori, E. , M. Baratti , A. Viviano , et al. 2024. “A Multidisciplinary Approach Unveils the Distribution of the Alpine Long‐Eared Bat *Plecotus Macrobullaris* (Vespertilionidae) in Italy.” Mammalia 88, no. 5: 445–450. 10.1515/mammalia-2024-0009.

[ece372308-bib-0038] Muscarella, C. , and A. Baragona . 2017. “The Endemic Fauna of the Sicilian Islands.” Biodiversity Journal 8, no. 1: 249–278.

[ece372308-bib-0039] Palombo, M. R. 2018. “Insular Mammalian Fauna Dynamics and Paleogeography: A Lesson From the Western Mediterranean Islands.” Integrative Zoology 13, no. 1: 2–20. 28688123 10.1111/1749-4877.12275 PMC5817236

[ece372308-bib-0040] Paradis, E. 2018. “Analysis of Haplotype Networks: The Randomized Minimum Spanning Tree Method.” Methods in Ecology and Evolution 9, no. 5: 1308–1317.

[ece372308-bib-0061] Rotolo, S. G. , S. Scaillet , S. La Felice , and G. Vita‐Scaillet . 2013. “A Revision of the Structure and Stratigraphy of Pre‐Green Tuff Ignimbrites at Pantelleria (Strait of Sicily).” Journal of Volcanology and Geothermal Research 250: 61–74. 10.1016/j.jvolgeores.2012.10.009.

[ece372308-bib-0041] Rozas, J. , A. Ferrer‐Mata , J. C. Sánchez‐DelBarrio , et al. 2017. “DnaSP 6: DNA Sequence Polymorphism Analysis of Large Datasets.” Molecular Biology and Evolution 34: 3299–3302. 10.1093/molbev/msx248. 29029172

[ece372308-bib-0042] Rutschmann, F. 2006. “Molecular Dating of Phylogenetic Trees: A Brief Review of Current Methods That Estimate Divergence Times.” Diversity and Distributions 12, no. 1: 35–48. 10.1111/j.1366-9516.2006.00210.x.

[ece372308-bib-0043] Salzburger, W. , G. B. Ewing , and A. Von Haeseler . 2011. “The Performance of Phylogenetic Algorithms in Estimating Haplotype Genealogies With Migration.” Molecular Ecology 20, no. 9: 1952–1963. 21457168 10.1111/j.1365-294X.2011.05066.x

[ece372308-bib-0044] Schofield, H. , G. Reiter , and S. E. Dool . 2023. “Lesser Horseshoe Bat *Rhinolophus hipposideros* (André, 1797).” In Chiroptera. Handbook of the Mammals of Europe, edited by D. Russo . Springer. 10.1007/978-3-030-44029-9_39.

[ece372308-bib-0045] Schrader, J. , I. J. Wright , H. Kreft , et al. 2024. “Trait Filtering in Island Floras: A Conceptual Framework.” Journal of Biogeography 51, no. 9: 1596–1606.

[ece372308-bib-0046] Smedmark, J. E. , T. Eriksson , and B. Bremer . 2010. “Divergence Time Uncertainty and Historical Biogeography Reconstruction–an Example From Urophylleae (Rubiaceae).” Journal of Biogeography 37, no. 12: 2260–2274. 10.1111/j.1365-2699.2010.02366.x.

[ece372308-bib-0047] Spatola, D. , A. Micallef , A. Sulli , et al. 2018. “The Graham Bank (Sicily Channel, Central Mediterranean Sea): Seafloor Signatures of Volcanic and Tectonic Controls.” Geomorphology 318: 375–389. 10.1016/j.geomorph.2018.07.006.

[ece372308-bib-0048] Spector, S. 2002. “Biogeographic Crossroads as Priority Areas for Biodiversity Conservation.” Conservation Biology 16, no. 6: 1480–1487.

[ece372308-bib-0049] Stöck, M. , G. Grifoni , N. Armor , U. Scheidt , A. Sicilia , and N. Novarini . 2016. “On the Origin of the Recent Herpetofauna of Sicily: Comparative Phylogeography Using Homologous Mitochondrial and Nuclear Genes.” Zoologischer Anzeiger‐A Journal of Comparative Zoology 261: 70–81.

[ece372308-bib-0050] Stöck, M. , A. Sicilia , N. M. Belfiore , et al. 2008. “Post‐Messinian Evolutionary Relationships Across the Sicilian Channel: Mitochondrial and Nuclear Markers Link a New Green Toad From Sicily to African Relatives.” BMC Evolutionary Biology 8: 1–19. 18294389 10.1186/1471-2148-8-56 PMC2276203

[ece372308-bib-0051] Stoffberg, S. 2007. Molecular Phylogenetics and the Evolution of High‐Frequency Echolocation in Horseshoe Bats (Genus Rhinolophus). Ph.D. Thesis in the Department of Zoology,. University of Cape Town, South Africa.

[ece372308-bib-0052] Storch, G. 1974. “Quartare Fledermaus‐Faunen von der insel Malta.” Senchenbergiana Lethaea 5: 407–434.

[ece372308-bib-0053] Suchard, M. A. , P. Lemey , G. Baele , D. L. Ayres , A. J. Drummond , and A. Rambaut . 2018. “Bayesian Phylogenetic and Phylodynamic Data Integration Using BEAST 1.10.” Virus Evolution 4: vey016. 10.1093/ve/vey016. 29942656 PMC6007674

[ece372308-bib-0054] Tamura, K. , G. Stecher , and S. Kumar . 2021. “MEGA11: Molecular Evolutionary Genetics Analysis Version 11.” Molecular Biology and Evolution 38: 3022–3027. 33892491 10.1093/molbev/msab120 PMC8233496

[ece372308-bib-0055] Templeton, A. R. , K. A. Crandall , and C. F. Sing . 1992. “A Cladistic Analysis of Phenotypic Associations With Haplotypes Inferred From Restriction Endonuclease Mapping and DNA Sequence Data. III. Cladogram Estimation.” Genetics 132: 619–633. 1385266 10.1093/genetics/132.2.619 PMC1205162

[ece372308-bib-0056] Troia, A. , F. M. Raimondo , and A. Geraci . 2012. “Does Genetic Population Structure of *Ambrosina Bassii* L.(Araceae, Ambrosineae) Attest a Post‐Messinian Land‐Bridge Between Sicily and Africa?” Flora‐Morphology, Distribution, Functional Ecology of Plants 207, no. 9: 646–653.

[ece372308-bib-0057] Vences, M. , A. Miralles , S. Brouillet , et al. 2021. “iTaxoTools 0.1: Kickstarting a Specimen‐Based Software Toolkit for Taxonomists.” Megataxa 6: 77–92. 10.11646/megataxa.6.2.1.

[ece372308-bib-0058] Vences, M. , S. Patmanidis , J. C. Schmidt , M. Matschiner , A. Miralles , and S. S. Renner . 2024. “Hapsolutely: a User‐Friendly Tool Integrating Haplotype Phasing, Network Construction and Haploweb Calculation.” Bioinformatics Advances 4: vbae083. 10.1093/bioadv/vbae083. 38895561 PMC11184345

[ece372308-bib-0059] Vodă, R. , L. Dapporto , V. Dincă , et al. 2016. “Historical and Contemporary Factors Generate Unique Butterfly Communities on Islands.” Scientific Reports 6, no. 1: 28828. 27353723 10.1038/srep28828 PMC4926222

[ece372308-bib-0060] Yule, G. U. 1925. “II. A Mathematical Theory of Evolution, Based on the Conclusions of Dr. J. C. Willis, F. R. S.” Philosophical Transactions of the Royal Society of London 213: 21–87.

