# Supplementary material for: Across the Channel and Through Time: Did Lesser Horseshoe Bats Colonise Pantelleria From Europe or North Africa?
Source: Ecol Evol. 2025 Oct 10;15(10):e72308. doi: 10.1002/ece3.72308 (PMC12514184; doi:10.1002/ece3.72308)
Supplement: Supplementary file 1 — Appendix S1: ece372308‐sup‐0001‐AppendixS1.docx. [file ECE3-15-e72308-s001.docx]

**Supplementary Material**

**Table S1**. Samples of *Rhinolophus hipposideros* used in this analysis, with origins, references and accession numbers.

| **Origin** | **Cytochrome-b** | **COI** | **12S** | **Reference** |
| --- | --- | --- | --- | --- |
| Albania | OP895179 | NA | NA | Benda et al., 2023 |
| Albania | OP895178 | NA | NA | Benda et al., 2023 |
| Albania | OP895177 | NA | NA | Benda et al., 2023 |
| Algeria | PV330063 | PV277618 | PV277973 | This work |
| Algeria | PV330064 | PV277618 | PV277973 | This work |
| Algeria | PV330065 | PV277618 | PV277973 | This work |
| Algeria | PV330066 | PV277618 | PV277973 | This work |
| Algeria | PV330067 | PV277618 | PV277973 | This work |
| Algeria | PV330068 | PV277618 | PV277973 | This work |
| Algeria | PV330069 | PV277618 | PV277973 | This work |
| Algeria | PV330070 | PV277618 | PV277973 | This work |
| Algeria | PV330071 | PV277618 | PV277973 | This work |
| Algeria | PV330072 | PV277618 | PV277973 | This work |
| Austria | KC978456 | NA | NA | Dool et al., 2013 |
| Bulgaria | KC978433 | NA | NA | Dool et al., 2013 |
| Bulgaria | KC978424 | NA | NA | Dool et al., 2013 |
| Bulgaria | KC978423 | NA | NA | Dool et al., 2013 |
| Bulgaria | KC978425 | NA | NA | Dool et al., 2013 |
| Bulgaria | KU531352 | NA | NA | Dool et al., 2016 |
| Central Italy | KC978381 | NA | NA | Dool et al., 2013 |
| Central Italy | KC978382 | NA | NA | Dool et al., 2013 |
| Central Italy | PV330032 | PV330031 | PV277983 | This work |
| Cyprus | OP895188 | NA | NA | Benda et al., 2023 |
| Cyprus | OP895185 | NA | NA | Benda et al., 2023 |
| Cyprus | KC978613 | NA | NA | Dool et al., 2013 |
| Czech Republic | KC978629 | NA | NA | Dool et al., 2013 |
| France | OQ885420 | OQ706679 | NA | Ruedi et al., 2023 |
| France | KC978713 | NA | NA | Dool et al., 2013 |
| France | KC978598 | NA | NA | Dool et al., 2013 |
| France | KC978599 | NA | NA | Dool et al., 2013 |
| France | KC978595 | NA | NA | Dool et al., 2013 |
| France | KC978593 | NA | NA | Dool et al., 2013 |
| France | KC978590 | NA | NA | Dool et al., 2013 |
| France | KC978588 | NA | NA | Dool et al., 2013 |
| France | KC978584 | NA | NA | Dool et al., 2013 |
| France | KC978529 | NA | NA | Dool et al., 2013 |
| France | KC978597 | NA | NA | Dool et al., 2013 |
| France | KC978532 | NA | NA | Dool et al., 2013 |
| France | KC978586 | NA | NA | Dool et al., 2013 |
| Georgia | NA | JF443130 | NA | Kruskop et al., 2012 |
| Georgia | NA | JF443131 | NA | Kruskop et al., 2012 |
| Ireland | KC978650 | NA | NA | Dool et al., 2013 |
| Ireland | KC978647 | NA | NA | Dool et al., 2013 |
| Ireland | KC978644 | NA | NA | Dool et al., 2013 |
| Ireland | KC978583 | NA | NA | Dool et al., 2013 |
| Ireland | KC978574 | NA | NA | Dool et al., 2013 |
| Ireland | KC978571 | NA | NA | Dool et al., 2013 |
| Ireland | KC978570 | NA | NA | Dool et al., 2013 |
| Ireland | KC978567 | NA | NA | Dool et al., 2013 |
| Ireland | KC978560 | NA | NA | Dool et al., 2013 |
| Ireland | KC978556 | NA | NA | Dool et al., 2013 |
| Ireland | KC978535 | NA | NA | Dool et al., 2013 |
| Ireland | KC978527 | NA | NA | Dool et al., 2013 |
| Jordan | OP895202 | NA | NA | Dool et al., 2013 |
| Kosovo | KC978607 | NA | NA | Dool et al., 2013 |
| Lebanon | OP895213 | NA | NA | Benda et al., 2023 |
| Lebanon | OP895212 | NA | NA | Benda et al., 2023 |
| Lebanon | OP895211 | NA | NA | Benda et al., 2023 |
| Lebanon | OP895210 | NA | NA | Benda et al., 2023 |
| Lebanon | OP895209 | NA | NA | Benda et al., 2023 |
| Lebanon | OP895208 | NA | NA | Benda et al., 2023 |
| Lebanon | OP895206 | NA | NA | Benda et al., 2023 |
| Lebanon | OP895205 | NA | NA | Benda et al., 2023 |
| Lebanon | OP895204 | NA | NA | Benda et al., 2023 |
| Lebanon | OP895203 | NA | NA | Benda et al., 2023 |
| Lebanon | KC978643 | NA | NA | Dool et al., 2013 |
| Lebanon | KC978642 | NA | NA | Dool et al., 2013 |
| Lebanon | KC978641 | NA | NA | Dool et al., 2013 |
| Lebanon | KC978638 | NA | NA | Dool et al., 2013 |
| Lebanon | KC978609 | NA | NA | Dool et al., 2013 |
| Malta | MN045625 | MN031866 | MN028644 | Mifsud and Vella, 2019 |
| Malta | MN045619 | MN031864 | MN028650 | Mifsud and Vella, 2019 |
| Malta | MN045618 | MN031860 | MN028651 | Mifsud and Vella, 2019 |
| Malta | MN045616 | MN031853 | MN028643 | Mifsud and Vella, 2019 |
| Malta | NA | NA | MN028649 | Mifsud and Vella, 2019 |
| Malta | NA | NA | MN028648 | Mifsud and Vella, 2019 |
| Malta | NA | NA | MN028647 | Mifsud and Vella, 2019 |
| Malta | KC978379 | NA | NA | Dool et al., 2013 |
| Malta | KC978378 | NA | NA | Dool et al., 2013 |
| Malta | KC978380 | NA | NA | Dool et al., 2013 |
| Morocco | OP895216 | NA | NA | Benda et al., 2023 |
| Morocco | OP895215 | NA | NA | Benda et al., 2023 |
| North Italy | KC978387 | FR856837 | NA | Galimberti et al., 2012; Dool et al., 2013 |
| North Italy | KC978388 | NA | NA | Dool et al., 2013 |
| North Italy | KC978716 | NA | NA | Dool et al., 2013 |
| Pantelleria | PV330073 | OR532431 | PV277966 | This work |
| Pantelleria | PV330073 | OR532432 | PV277966 | This work |
| Pantelleria | PV330073 | OR532433 | PV277966 | This work |
| Pantelleria | PV330073 | OR532434 | PV277966 | This work |
| Pantelleria | PV330073 | OR532435 | PV277966 | This work |
| Pantelleria | PV330073 | OR532436 | PV277966 | This work |
| Pantelleria | PV330073 | OR532437 | PV277966 | This work |
| Portugal | NA | MT407329 | NA | This work |
| Portugal | NA | MT407328 | NA | This work |
| Slovakia | OP895222 | NA | NA | Benda et al., 2023 |
| Slovakia | OP895221 | NA | NA | Benda et al., 2023 |
| Slovakia | KC978632 | NA | NA | This work |
| Slovakia | KC978628 | NA | NA | This work |
| Slovakia | KC978627 | NA | NA | This work |
| Slovenia | KC978470 | NA | NA | This work |
| Slovenia | KC978468 | NA | NA | This work |
| Slovenia | KC978466 | NA | NA | This work |
| Slovenia | KC978460 | NA | NA | This work |
| Southern Italy | KC978389 | NA | NA | This work |
| Southern Italy | KC978391 | NA | NA | This work |
| Southern Italy | KC978392 | NA | NA | This work |
| Southern Italy | KC978393 | NA | NA | This work |
| Southern Italy | KC978390 | NA | NA | This work |
| Southern Italy | NA | PP808505 | NA | This work |
| Switzerland | NA | OQ706680 | NA | This work |
| Syria | OP895225 | NA | NA | Benda et al., 2023 |
| Syria | OP895224 | NA | NA | Benda et al., 2023 |
| Syria | OP895223 | NA | NA | Benda et al., 2023 |
| Syria | KC978620 | NA | NA | This work |
| Syria | KC978614 | NA | NA | This work |
| Turkey | OP895227 | NA | NA | Benda et al., 2023 |
| Turkey | OP895228 | NA | NA | Benda et al., 2023 |
| Turkey | KC978711 | NA | NA | This work |
| Turkey | KC978631 | NA | NA | This work |
| UK | KC978705 | NA | NA | This work |
| UK | KC978704 | NA | NA | This work |
| UK | KC978700 | NA | NA | This work |
| UK | KC978699 | NA | NA | This work |
| UK | KC978687 | NA | NA | This work |
| UK | KC978674 | NA | NA | This work |
| UK | KC978673 | NA | NA | This work |
|  |  |  |  |  |


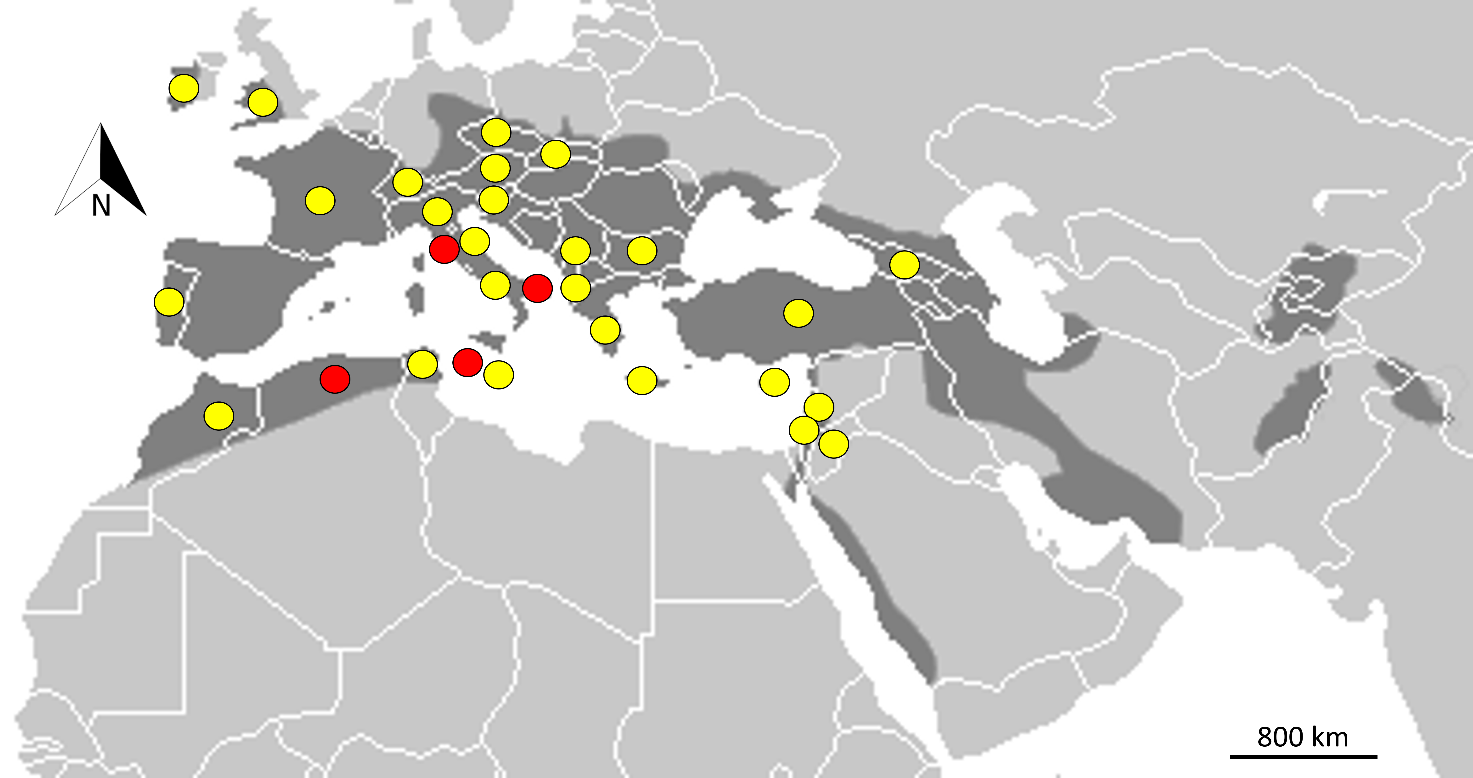


**Figure S1**. Distribution map of *Rhinolophus hipposideros* (shaded area: see [www.iucnredlist.org](http://www.iucnredlist.org). Accessed on 19.08.2025), showing sampling sites of newly collected individuals (yellow circles) and sequences deposited in GenBank (red circles).


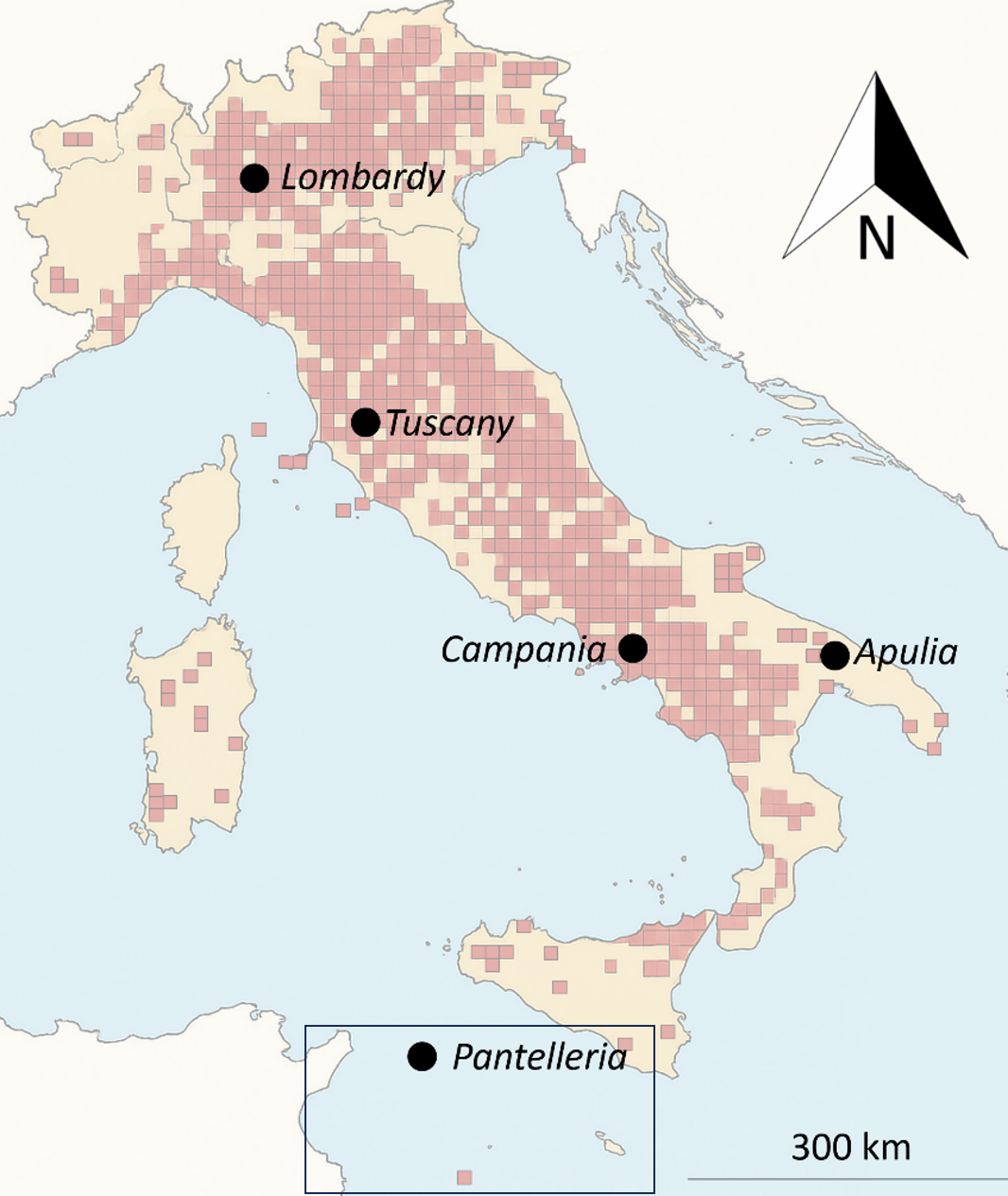


**Figure S2**. Distribution of *Rhinolophus hipposideros* in Italy and location of sampling sites (black dots). The inbox highlights the Sicilian channel.
